# Leptospirosis in Campinas, Brazil: The interplay between drainage, impermeable areas, and social vulnerability

**DOI:** 10.1371/journal.pntd.0013560

**Published:** 2025-09-29

**Authors:** Thiago Salomão de Azevedo, Shahista Nisa, Stuart Littlejohn, Renata L. Muylaert

**Affiliations:** 1 Secretary of Health, Municipality of Santa Barbara d’Oeste, Sao Paulo, Brazil; 2 Department of Biodiversity, Institute of Biosciences, UNESP, Rio Claro, Sao Paulo, Brazil; 3 School of Veterinary Science, Massey University, Palmerston North, New Zealand; 4 Sydney School of Veterinary Science, The University of Sydney, Sydney, Australia; The University of Tennessee Health Science Center, United States of America

## Abstract

Leptospirosis is an epidemic disease caused by bacteria of the *Leptospira* genus. Its risk is closely associated with inadequate sanitation and flooding, a common public health challenge in large urban centers together with urban environmental modifications, and socio-economic factors. This retrospective observational research investigated the association between the distribution of leptospirosis cases and three contextual factors, drainage, soil impermeability and social vulnerability in Campinas city, São Paulo, Brazil. We hypothesized that the number of cases will increase in areas that are impermeable and in proximity to drainage systems as well as where social vulnerability is high. We investigated the associations based on 86 autochthonous cases, comparing cases where infection risk was linked to contact with floodwater or mud (n = 54) to cases associated with other exposures (n = 32). Spatial statistics were used to map disease distribution and investigate the relationship between leptospirosis cases and contextual factors. Our results indicate that leptospirosis cases density rises near drainage systems, peaking at 200 m. Risk is elevated in socially vulnerable areas, particularly where floodwater or mud exposure is high, and in highly impermeable areas. This study demonstrated that leptospirosis risk remains highly determined by living and working conditions. These findings support targeted strategies to deliver effective prevention, treatment and control interventions in highly populated urban areas of the Global South and similar contexts. Furthermore, combining local contextual environmental information with spatial analysis produces relevant evidence for guiding health public policy and spatial planning and provides precise parameters for future epidemiological models and prevention actions.

## Author summary

This study explores the links between environmental and contextual factors that influence the risk of leptospirosis transmission to humans in Campinas, São Paulo, Brazil. Leptospirosis is an infectious disease caused by the *Leptospira* bacteria. We investigated how drainage systems, impermeable soil area, and social vulnerability contribute to disease distribution, mapping spatial conditions for 86 cases associated with water and mud contact and other risks. Our findings highlight how environmental and socio-economic factors intersect to influence public health, shedding light on the role of urban planning and drainage infrastructure in the transmission risk of infectious diseases. Our approach reveals that flood-related transmission risk is highest within 200 meters of drainage systems and in socially vulnerable, highly impermeable areas. These findings provide actionable insights for urban planners and public health officials, demonstrating how targeted infrastructure improvements and flood management can potentially reduce disease risk in the most affected communities. The study contributes to practical parameters for epidemiological modeling and offers a replicable methodology for addressing climate-sensitive diseases in rapidly urbanizing areas of the Global South.

## Introduction

Public health in urban areas faces different hazards associated with suboptimal urban planning, social vulnerability and climate-related hazards [1]. A hazard that is likely to increase is flooding, an extreme phenomenon that severely impacts human populations. Flooding increases the risk of infectious and non-communicable diseases, including leptospirosis [2, 3], hepatitis [4], mental illness and heart problems [5]. Leptospirosis has been recognized as an infection of intensifying global public health concern and particularly in the Global South [6], yet it remains a neglected tropical disease [7]. Annually, more than one million symptomatic cases, resulting in at least 60,000 deaths, are reported worldwide [8]. The symptoms of leptospirosis in humans are usually non-specific, leading to frequent misdiagnosis or delays in diagnosis. The classic presentation includes fever, chills, severe headache, muscle aches, conjunctival suffusion, nausea, and vomiting [9]. Severe forms of leptospirosis are Weil’s disease, with jaundice and renal insufficiency or interstitial alveolar hemorrhage, congestion, and edema, and pulmonary hemorrhagic syndrome, which is a severe lung condition [10, 11]. The disease causes a heavy health burden in tropical regions, particularly in areas characterized by poverty, humid climate, and lack of adequate health infrastructures [12].

Leptospirosis is caused by infection from bacteria of the genus *Leptospira*, which can infect land and sea mammals [13], including domestic animals, wildlife, and humans [14]. Human infections occur from direct contact with infected urine and excretions by mammals [15] or indirectly through the contaminated environment [16]. The greatest risk factor for human infection is exposure to contaminated water [17]. Water offers a suitable medium for leptospires, agglutinated with organic matter, to remain infectious for long periods of time [14]. This risk is amplified in urban settings, where high population density and unpredictable flooding can increase exposure and hinder effective protection. Rats are the main hosts in urban settings which, combined with water, creates ideal conditions for leptospirosis transmission as leptospires can survive in water and humid soil [18]. Occupation and poverty can increase leptospirosis risk in areas where there is pervasive bad sanitation, rodent infestation, flooded soils, and lack of access to healthcare. However, the precise magnitude and combined effects of these factors remain unknown for many cities. For instance, exposure may vary with social vulnerability and geographical location, underscoring the need for studies that map cases together with social and environmental exposure factors. Such quantified data can help allocate surveillance and prevention efforts in urban infrastructure.

Recent decades have seen an increase in the incidence of leptospirosis in urban areas prone to inundation [19]. Many cities are designed and built in proximity with water bodies because the resources these environments provide are essential for population livelihood, particularly during city formation [20]. However, several anthropic activities that promote contact with these sites lead to changes in the biological cycles of disease vectors, hosts and reservoirs, as well as in the ways humans are exposed to them [21]. In Brazil, the growing urbanization in large cities contributes to leptospirosis outbreaks because the concentration of human habitat generates residues that can serve as refuge and provide resources for rodents [3]. In addition, the increase in urban sprawling and paved roads and streets cause soil impermeability and prevent or hinder the infiltration of rainwater, resulting in flooding and a greater number of infected people [20, 22]. Populations of low socioeconomic status are more affected by leptospirosis due to the synergistic effect of factors such as low-quality sanitation services, environmental and housing characteristics and work activities that often favor contact with contaminated water or soil [23,24].

In geomorphology, drainage systems, also known as river systems, refer to the structural patterns formed by streams, rivers, and lakes within a drainage basin. Because of gravity and variations in soil permeability, drainage patterns range from dendritic or fractal to parallel and rectangular. These patterns depend highly on the topography and geology of the land, and include artificial alterations. In urban settings, drainage involves either the natural or artificial removal of surface and subsurface water from an area with excess water through ditches, plantings, drain gutters and channeled rivers. In such settings, we expect that the ideal condition for leptospirosis transmission via contaminated water during a flood happens when two conditions are met. First, areas will be closer to drainage systems and second, these areas will have high soil impermeability.

As a thorough understanding of the ecology of leptospirosis in relation with urban drainage and social vulnerability are important for the control of outbreaks, this study utilizes Geographic Information Systems (GIS) tools and spatial analysis to identify the geographical factors associated with leptospirosis occurrence in Campinas, São Paulo, Brazil. We hypothesize that areas with critical flood points have a higher frequency of leptospirosis cases, as they meet two key flood-prone risk conditions: proximity to drainage systems and high levels of impermeability to water. Additionally, we expect a negative correlation between leptospirosis incidence and socioeconomic status, with cases concentrated in areas of greater social vulnerability. To test this hypothesis, we focus on cases where infection risk is linked to contact with floodwater or mud, comparing them to cases associated with other exposures. Our primary objective was to quantify spatial patterns of the disease, delineate high-risk areas, and assess environmental factors contributing to its spread. By doing so, we aim to support epidemiological surveillance efforts and enhance the response to health emergencies related to leptospirosis.

## Methods

### Study area

The municipality of Campinas is located in the interior of São Paulo State with an estimated population of 1,139,047 inhabitants and a population density of 1,433.541 hab/km² [23]. The climate is characterized as Tropical at altitude (Cwa) according to the Koppen/ Geiger classification [24]. The annual temperature average is 20.7 °C, with dry and mild winters and a long, rainy summer [25].

### Leptospirosis data

The incubation period for leptospirosis ranges from two to 30 days, according to the Brazilian Health Surveillance Manual. The notification form of the Information System for Notifiable Diseases (SINAN) [26] for leptospirosis contains a section in which the risk factors for the disease are primarily associated with the environment and the patients’ exposure conditions [27]. The main risk factors in the Additional Case Data section are listed in Table 1.

**Table 1:** Associated risks reported in the notification form in the Information System for Notifiable Diseases (SINAN) for leptospirosis in Brazil.

Risk	Description
Risk related to floodwater or mud	Exposure to floodwater or mud from floods is one of the primary risk factors for infection.
Contact with rodents	Direct contact with rodents or their excrement and urine is a common form of leptospirosis transmission and the presence of rodent signs in areas where the patient lives or works is a significant risk factor, as these animals can transmit the bacteria through their urine.
Vacant lots	Contact with undeveloped or abandoned land can be a source of infection due to the presence of rodents and other animals.
Contact with garbage	Handling waste or working in places with accumulated trash can also be a risk situation.
Animal husbandry	The presence of domestic or farm animals (such as pigs, cows, etc.) that can be hosts of leptospirosis is a risk factor.
Cleaning water tanks	Exposure to untreated water from water tanks can be a source of contamination.
Exposure to sewage	Contact with sewage, which may be contaminated with the leptospirosis bacteria, is also a risk situation.
Contact with grain storage	Contact with stored grains that may have been contaminated by rodents or other animals.
Exposure to bodies of water	Swimming or coming into contact with rivers, lakes, or other bodies of water that could be contaminated.
Contact with crops	Contact with harvested crops or stored grains that may have been contaminated by rodents or other animals.
Others	Other risks not defined previously.

These risk factors are recorded in the notification form and help public health authorities investigate the origin of cases and identify high-risk areas for leptospirosis infection, allowing for more effective preventive measures. Thus, patients with well-defined symptoms who had contact with any risk factor within this time window were considered target cases in our retrospective observational study.

The dataset of autochthonous leptospirosis cases recorded between 2007 and 2010 was provided by the Campinas Municipal Health Department in an anonymized form and aggregated by city blocks through a numeric identifier. This identifier was linked to the municipal cadastral map, which contained the corresponding codes, allowing the integration of the leptospirosis case database with the official urban grid. The dataset included only the following variables: block identifier, associated risk factor, date of first symptoms, indication of autochthony, and the type of laboratory test performed (ELISA-IgM or Microscopic Agglutination). No personal information, such as age, sex, or whether the probable site of occurrence corresponded to the patient’s residence, was provided. Given these characteristics, and in accordance with Brazilian Law No. 13.709/2018 (General Data Protection Law – LGPD), the data are classified as aggregated and anonymized health information; therefore, ethics committee approval was not required for this study.

The geocoding process was carried out using the official municipal cadastral map provided by the local government. The centroid coordinates of each block were extracted in the UTM coordinate system (Zone 23, SIRGAS 2000 Datum) and used as a proxy for the probable site of infection. Cases lacking sufficient spatial identification, specifically the cadastral block code for the probable site of infection, were excluded from the analysis as they could not be georeferenced within the municipal cadastral database. The yearly number of cases from 2001 to 2023 were incorporated to reveal disease dynamics across time based on SINAN data [26]. Yearly leptospirosis cases were plotted using R 4.4.1 [28] and *ggplot* [29].

### Spatial distribution of cases and environmental critical points

The verification of rainfall data and anthropogenic variables in the urban area of Campinas, São Paulo, was carried out based on the application of a sensitivity and spatial analysis. Standardized regression coefficients (SRC) and regression models were applied to assess the impact of these variables on leptospirosis cases. A high absolute value for the coefficient indicates that the input variable has a strong influence on leptospirosis risk. An SRC close to zero suggests that the variable has little influence on the model. To perform this procedure, a spatial database containing the following variables was first created: accumulated precipitation on the day that caused the flood event, the São Paulo social vulnerability index (IPVS), and the impermeable area index. Then, we calculated the distance of cases to the closest drainage system, to quantify the spatial relationship between disease occurrence and drainage. We defined drainage as any area of land where precipitation falls and moves to a common outlet. As a data source for drainage network data were obtained from the Municipal Basic Sanitation Plan, which includes the official digital cartographic database. The files were provided in shapefile format with the same reference system (UTM Zone 23, SIRGAS 2000 Datum), ensuring consistency with the case data. The original scale of these data was 1:10,000, which is appropriate for environmental analyses in urban areas [30]. This file was imported into QGIS v3.34.11 [31], where a euclidean distance map was compiled. The georeferenced leptospirosis cases were overlaid. Following this procedure, the distance values of these cases relative to the drainage network were extracted.

Rainfall data were obtained from the Hydrological Database of the Department of Water and Energy of the State of São Paulo (DAEE), through 10 meteorological stations. These stations were chosen because they had continuous daily rainfall data. After inspecting the values from the rain gauges, the flood events in the city of Campinas - SP, from 2007 to 2010, were validated through the flood database of the Civil Defense of the Municipality of Campinas. The concept of flooding used here is defined as the overflow of bodies of water (rivers, streams, and lakes) due to heavy rains or an increase in water volume, which exceeds the natural limits of the riverbeds, flooding areas that are normally dry. Floods can cause significant damage to residences, infrastructure, and public safety. In urban areas, they occur where the drainage infrastructure is unable to handle large volumes of water, often due to soil impermeabilization [32]. This makes information on rain, drainage and permeability of surfaces relevant to understand disease risk for leptospirosis. Using the dates of occurrence of this type of natural disaster, the precipitation values were organized in a database.

This information was associated with the geographic coordinates of the rain gauges and, through the use of the minimum curvature interpolation method, four maps of accumulated precipitation were compiled, referring to the days on which flood events were recorded in the Campinas region during the study period (S1 Fig). This method was used because it has a superior performance when compared to other interpolation methods [33].

A specific social-demographic index titled São Paulo Social Vulnerability Index (IPVS), which is based on the spatial identification of the areas of vulnerability of the resident population, classifies the urban census sectors into six categories, which range from very low or no vulnerability to very high vulnerability [34]. This index considers a range of factors influencing vulnerability, including: income, employment status, age distribution, gender, household structure, access to education, healthcare, housing, and the existence of community development projects.

Finally, the impermeable area index was calculated using the normalized difference built-up index (NDBI) derived from a Landsat TM5 satellite image from 2010 (reference ID: LT52190762010108CUB01 - April 2010) with a 30-meter spatial resolution. This image was acquired through a search on the image service offered by the United States Geological Survey [35]. This index was developed from information found in [36]. The NDBI is expressed by the following equation:


NDBI=(MIR−NIR)/(MIR+NIR)(3)\]

Where: MIR = mid-infrared (Band 5)

NIR = near-infrared (Band 4)

We then performed an analysis associating cases of leptospirosis with the census tracts of the municipality to verify the density of cases in each IPVS category, from extremely low to very high levels of vulnerability.

NDBI quantifies the impermeability of surfaces in a given area, considering materials such as asphalt, concrete, roofs, and other artificial covers that prevent water infiltration into the soil. This index exhibits a certain level of homogeneity, particularly in the urban center of Campinas, where its variation is limited. Therefore, for NDBI, we used the raw number of cases to examine the relationship between impermeability levels and post-flood leptospirosis risk as using case density based on NDBI could distort the identification of critical areas, as this index is not area-corrected. In contrast, for IPVS, we used case density as the response variable to indicate post-flood leptospirosis risk. Correlating case density with IPVS allows for a proportional identification of higher-risk areas, as vulnerability classes represent regions of different sizes and socioeconomic characteristics that influence exposure and interactions with the environment. All maps were developed in QGIS v3.34.11 [31], using the coordinate reference system UTM Zone 23S, SIRGAS 2000 datum. The final cartographic products were standardized at a scale of 1:280,000 to ensure spatial comparability.

To evaluate the relationship between leptospirosis cases and environmental indices (distance from urban drainage, impervious surfaces, and social vulnerability), we applied second-degree polynomial regression models (quadratic fits) separately for cases associated with floodwater/mud exposure and for other exposure risks. Polynomial regression was chosen to capture potential non-linear patterns while maintaining the linear structure of the coefficients. The regression curves were fitted during the graph compilation process in Microsoft Excel 2016, which computes polynomial coefficients using the Ordinary Least Squares (OLS) method. The resulting equations and coefficients of determination (R²) were obtained directly from the Excel trendline outputs. For each model, 95% confidence intervals (CIs) were estimated based on the correlation between the Waterproof Index and case frequencies. Pearson’s correlation coefficients (r) were calculated in Excel, and 95% CIs were derived using Fisher’s z-transformation. Confidence intervals were displayed as shaded bands around the fitted curves to illustrate the uncertainty of predicted mean values.

In addition, simple non-linear regressions were performed to examine the association between the probable sites of leptospirosis infection (classified according to flood-related risk and other risk factors, used as the response variable) and each explanatory variable: distance to the nearest drainage system, social vulnerability (IPVS), and impervious area (NDBI). These analyses were conducted in Microsoft Excel 2016, using the regression analysis module available in the *Data Analysis ToolPak add-in*.

## Results

An overview of confirmed leptospirosis cases in residents of Campinas between 2001–2023 revealed outbreaks consistent throughout the last decade. There were consistent outbreaks, with peaks in 2009, 2011, 2012, and 2013 (all exceeding 40 cases per year, S2 Fig). After 2016, the total number of cases declined consistently, never surpassing 11 cases per year. The total number of resident cases summed 502 cases, of which 105 occurred during our investigated period (2007–2010). From these, 51 did not report contact with floodwater or mud (‘other risk’ category), while 54 did.

**Fig 1:**
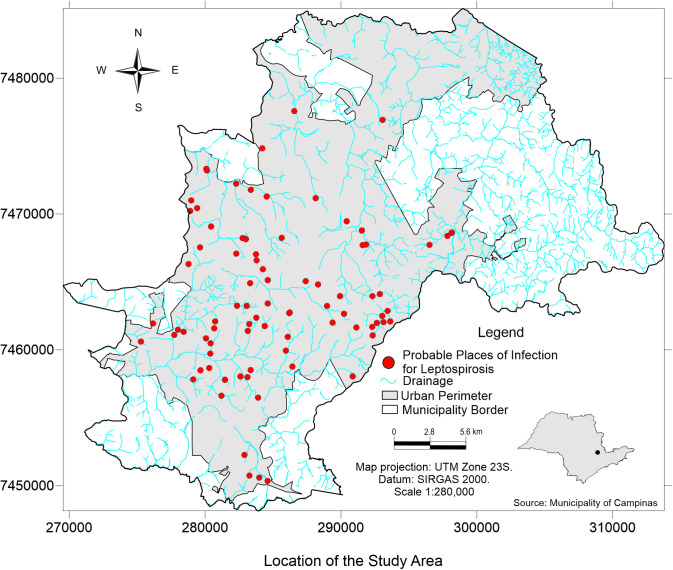
Spatial distribution of autochthonous leptospirosis cases in humans from 2007 to 2010 identified in Campinas, southeastern Brazil. Vector data (shapefile) from the Prefeitura Municipal de Campinas, as part of the city’s open data initiative. Administrative limits: https://informacao-didc.campinas.sp.gov.br/exporta_shp.php?id=119 (accessed March 2025). Streams: https://informacao-didc.campinas.sp.gov.br/exporta_shp.php?id=37 (accessed March 2025). The data is free of licences complying with the Transparência Pública Brasil (https://www.gov.br/cgu/pt-br/centrais-de-conteudo/campanhas/integridade-publica/transparencia-publica). Note that the data comes from Brazilian Public websites that may limit access to IP addresses outside of Brazil.

These 86 autochthonous cases (Fig 1) were further stratified by the environment and risk exposure conditions (19 cases that reported other exposures had incomplete information). This Fig 1 illustrates the localization of the probable infection sites of leptospirosis cases.

**Fig 2:**
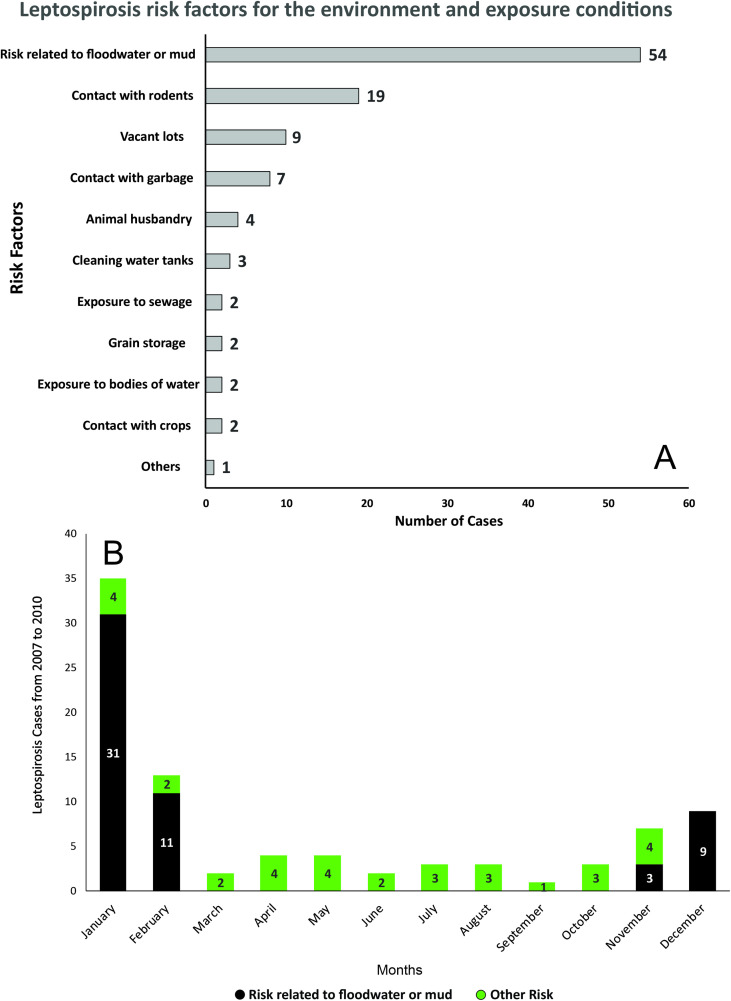
A. Exposures reported by leptospirosis cases and B. monthly distribution of leptospirosis cases (2007-2010) in Campinas, southeastern Brazil.

Fig 2A presents the main exposures associated with the leptospirosis cases during the study period. The most significant factor was the risk related to floodwater or mud, which accounted for 51% (n = 54) of the cases, while other risks represented 49% (n = 51) of the cases. Contact with rodents was the second major risk factor, with 18.1% (n = 19) cases, reinforcing the connection between leptospirosis and the presence of rats, which are the main vectors of the *leptospira* bacteria. Other risk factors include vacant lots with 8.6% (n = 9) and contact with garbage representing 6.7% (n = 7), suggesting that inadequate urban conditions may increase the risk of infection. Occupational exposure factors, such as animal farming and cleaning water tanks, account for 3.8% (n = 4) and 2.9% (n = 3), respectively. Factors such as grain storage, contact with crops, exposure to sewage, and bodies of water each accounted for 1.9% of cases (n = 2 per category), indicating that while they are potential sources of contamination, they are less frequent in the analyzed population. The category labeled “Others” had only one recorded case.

Fig 2B shows the monthly distribution of leptospirosis cases, with the two selected categories: Risk related to floodwater or mud and other risks. January accounted for the highest number of leptospirosis cases, with 31 cases related to floodwater or mud. This is likely linked to heavy rains and floods that were typical for this period. In February, the number of cases related to floods decreased but remains the second highest month with 11 cases. December also shows an increase in flood-related cases, with 9 cases. In other months, the number of cases linked to floods and other risks was low, with only a few cases recorded from March to November.

**Fig 3:**
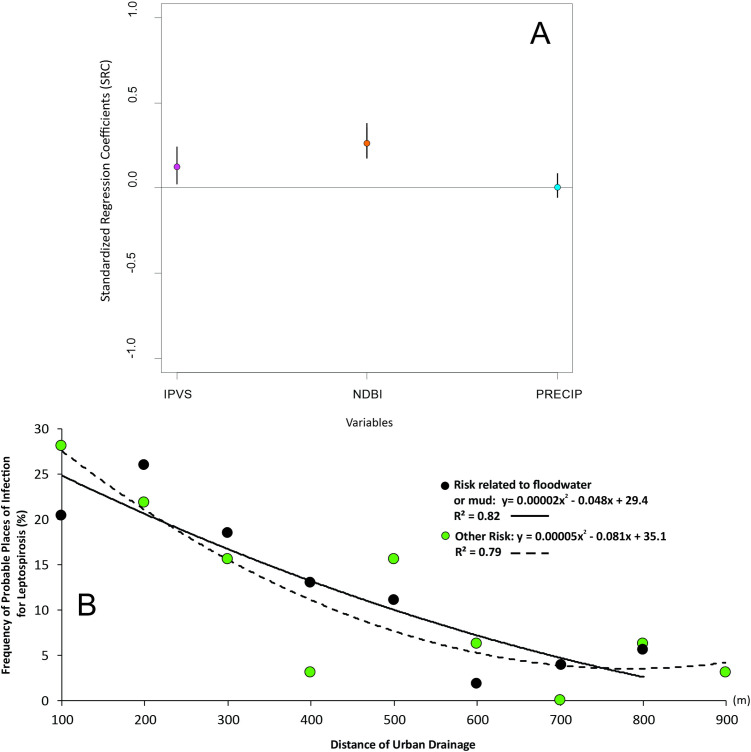
A. Sensitivity analysis of the São Paulo Social Vulnerability Index (IPVS), the Normalized Difference Built-up Index (NDBI), and precipitation, in relation to the number of leptospirosis cases associated. Positive SRC values indicate that increases in the variable are associated with a higher number of leptospirosis cases, whereas values near zero suggest little influence on case occurrence. B. Relationship between the frequency of leptospirosis cases and distance from urban drainage.

The sensitivity analysis revealed a weak relationship observed between the number of cases and the amount of rainfall, suggesting that flooding, rather than rainfall alone, increases the risk of infection. However, positive associations between IPVS and NDBI were observed (Fig 3A). The closer a location was to drainage systems, the higher the number of leptospirosis cases. This pattern holds true regardless of the specific mode of exposure (e.g., water/mud contact vs. other risks), as the adjusted curves for both exposure types largely overlap (Fig 3B, S1 Table). Additionally, a linear relationship was observed between the frequency of leptospirosis cases and their proximity to urban drainage systems (Fig 3B). As the distance increases, the frequency of infections strongly decreases (R² = 0.82), suggesting that leptospirosis is more concentrated near urban drainage systems, with the peak number of cases recorded at 200 m. The frequency of infections related to other risks also shows a decreasing trend but with a slightly weaker correlation (R² = 0.79) compared to flood risk. For flood-related cases, the fitted curve is steeper, suggesting a greater impact of these factors on the risk of infection. At distances greater than 700 meters, the difference between the two types of risk decreases, suggesting that, far from urban drains, other factors may influence the infection.

**Fig 4:**
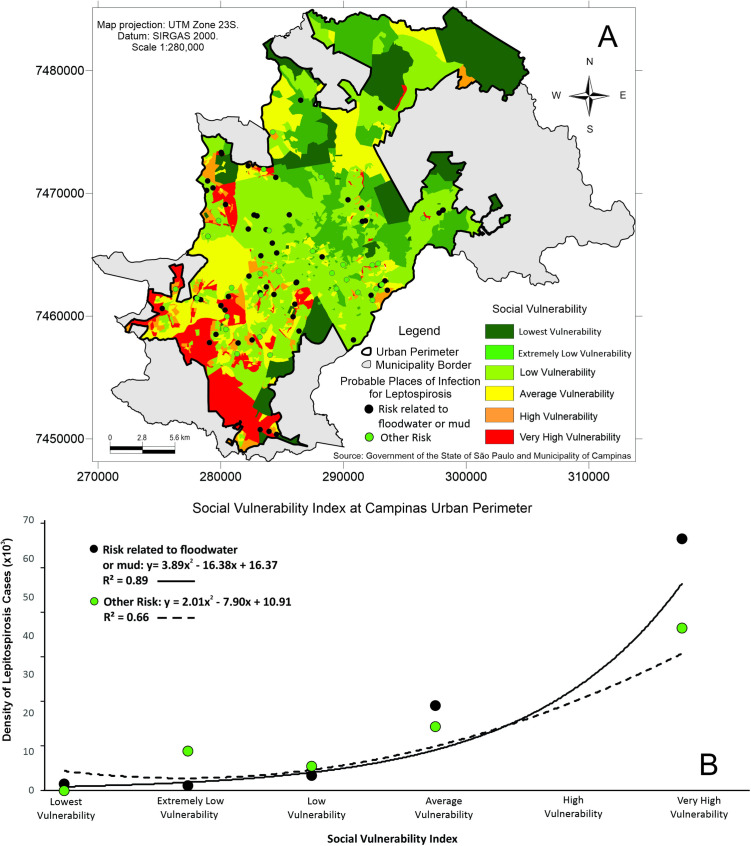
Higher social vulnerability is strongly linked to increased leptospirosis risk in Campinas, SP, Brazil. **A.** Spatial distribution of all human leptospirosis cases who reported contact with floodwater/mud and other kinds of exposure overlaid an index that classifies census tracts into clusters of the poorest areas, detailing living conditions and the location of the most social vulnerability (IPVS Index), from 2007 to 2010 period in Campinas, São Paulo, southeastern Brazil. **B.** Density of leptospirosis cases who reported contact with floodwater/mud and other kinds of exposure from 2007 to 2010 according to social vulnerability levels. R² represents the proportion of variance in the response variable explained by the covariate, where values closer to 1 indicate a better fit. Vectorial data (shapefile) source from the São Paulo State Government SEADE, available at: http://ipvs.seade.gov.br/view/index.php (accessed March 2025). The data is free of licenses complying with the Transparência Pública Brasil (https://www.gov.br/cgu/pt-br/centrais-de-conteudo/campanhas/integridade-publica/transparencia-publica). Note that this data comes from Brazilian Public websites that may limit access to IP addresses outside of Brazil.

**Fig 5:**
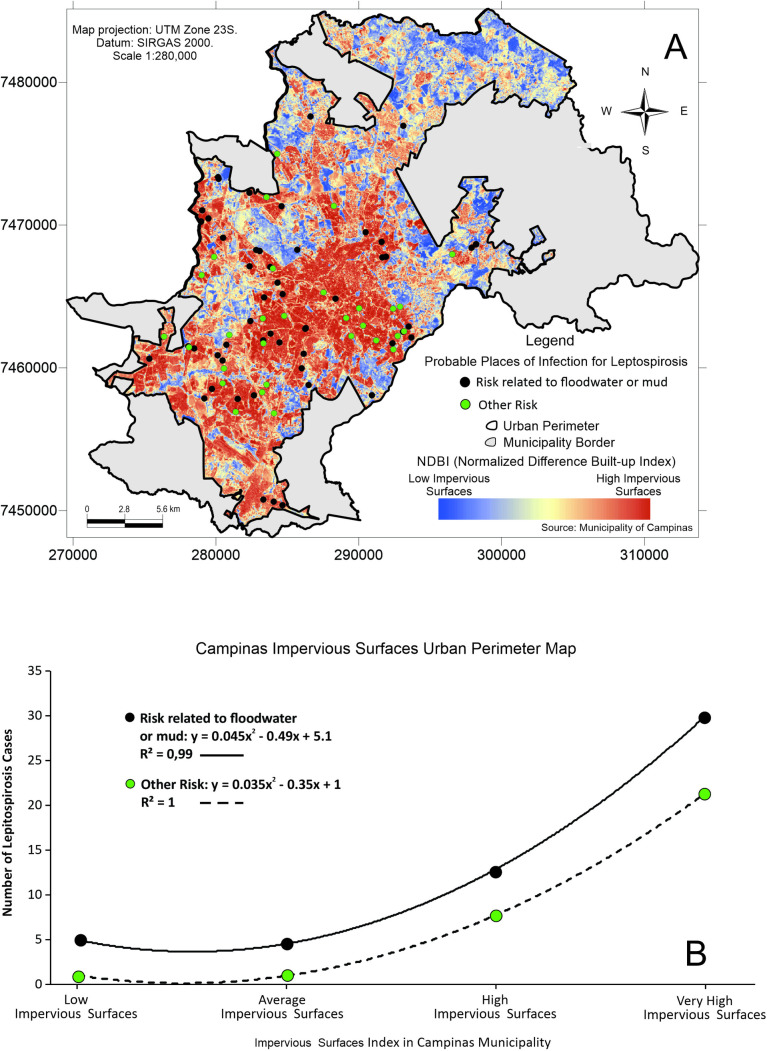
Leptospirosis cases are consistently higher in areas with more impervious surfaces. **A.** Spatial distribution of all human leptospirosis cases who reported contact with floodwater/mud and other kinds of exposure from 2007 to 2010 overlaid with ground permeability in Campinas, São Paulo, southeastern Brazil. **B.** Number of leptospirosis cases who reported contact with floodwater/mud and other kinds of exposure from 2007 to 2010 according to the impervious surfaces index. Vector data (shapefile) from the Prefeitura Municipal de Campinas, part of the city’s open data initiative. Administrative limits: https://informacao-didc.campinas.sp.gov.br/exporta_shp.php?id=119 (accessed March 2025). The data is free of licenses complying with the Transparência Pública Brasil (https://www.gov.br/cgu/pt-br/centrais-de-conteudo/campanhas/integridade-publica/transparencia-publica). Note that this data comes from Brazilian Public websites that may limit access to IP addresses outside of Brazil.

The spatial distribution of leptospirosis cases overlaid with IPVS illustrates that cases are concentrated in areas of higher social vulnerability (Fig 4A), with a positive relationship between the density of leptospirosis cases and increasing levels of social vulnerability (Fig 5B).

In the most vulnerable areas, cases linked to floodwater/mud exposure outnumbered cases from other exposures, suggesting that flooding-related contamination disproportionately affects the most vulnerable populations (Fig 4). As the IPVS increases (indicating higher social vulnerability), the density of leptospirosis cases related to floodwater/mud exposure shows a marked increase (Fig 4B, S2 Table). The relationship is non-linear and exhibits a steep rise, especially in regions with higher vulnerability, implying that communities with greater social vulnerability are more likely to be exposed to flood-related leptospirosis. The curve suggests that as vulnerability increases, flood-related risks and thus leptospirosis cases significantly increase as well, with a strong correlation (R² = 0.88). The density of leptospirosis cases attributed to other risks (such as rodent contact or poor urban conditions) also increases with higher vulnerability, but the trend is more gradual, indicating that other social determinants, like sanitation or living conditions, contribute to the risk of leptospirosis, but at a slower rate compared to the flood-related risk. This correlation is slightly weaker than that of flood-related cases (R² = 0.64), suggesting that although vulnerability impacts both types of risks, the connection is stronger for cases related to floods.

The spatial distribution of human leptospirosis cases in relation to ground permeability in Campinas shows that cases predominantly occurred in areas with highly impermeable grounds (Fig 5A). This emphasizes the role of urban environmental characteristics in disease transmission, likely due to the accumulation of floodwater and subsequent exposure risk (Fig 5B, S3 Table). However, unlike the drainage-related cases, the two exposure curves (floodwater/mud *versus* other risks) do not overlap but follow the same trend, with floodwater/mud exposure consistently leading to more cases. As the extent of impervious surfaces increases, the number of leptospirosis cases associated with floodwater and mud exposure rises significantly. The relationship is non-linear, showing a sharp upward trend as the proportion of impervious surfaces becomes higher. This indicates that areas with greater urban impermeabilization experience more flood-related leptospirosis cases, suggesting that even in better-drained environments, flooding events can still expose individuals to infection risk. The correlation is strong (R² = 0.87), showing that higher levels of impervious surfaces are strongly associated with a concentration of flood-related leptospirosis cases. The number of cases in the ‘other risk’ category also increases with greater impervious surface coverage, but the trend is less steep. This indicates that while more leptospirosis cases attributed to other factors (e.g., rodent exposure, unsanitary conditions) occur in areas with higher imperviousness, the increase is less pronounced than that observed for flood-related cases.

## Discussion

Here, we discuss insights from our findings around leptospirosis cases and temporal trends in Campinas, and discuss its relevance for global combat to leptospirosis through urban planning and social development. There is a clear connection between leptospirosis cases and social vulnerability which underpin inequalities in access to services, infrastructure quality and exposure to environmental hazards. Leptospirosis risk was amplified post-flooding, particularly for cases residing in areas of high social vulnerability and close to highly impermeable areas, when compared to cases who did not report exposure to floodwater or mud. In our analysis, we quantified these connections, highlighting a disproportionate increase in leptospirosis risk for vulnerable conditions.

The monthly variation of cases show that flood-associated leptospirosis cases are more prevalent during the rainy season and flood periods, with peaks in January and February, and a slight increase in cases in December (Fig 2B). On the other hand, cases associated with other risks seem to be less seasonal and more evenly distributed throughout the year, indicating that these may have a more dispersed distribution in the urban environment. While this pattern is expected given leptospirosis’ waterborne nature, our analysis provides a formal quantification of these dynamics, reinforcing the clear trends with empirical data. This not only validates common knowledge but also offers a measure of the interplay between flooding and disease risk.

Our estimated regression curves are presented in Figs 3B, 4B, and 5B. We identified a clear decrease in the probable location of leptospirosis infection as the distance from drainage increases, with the peak in cases observed at 200 m (Fig 3B). This is likely due to floodwaters accumulating closer to overflowing drains, increasing the risk of exposure to the *Leptospira* bacteria. This peak makes sense from the point of view of rodent host habitat and movement behavior. Rodents are associated with roosts around dirt banks close to drainage and sewer, likely 3x more common to be found in areas of bare soil or mixed ground [37], and those areas are likely to be right next to paved/closed drainage (proximity to drainage = 0) in our context.

As impervious surfaces increase, leptospirosis cases also rise, indicating that reduced soil permeability favors the accumulation of contaminated rainwater, contributing to outbreaks of the disease (Fig 5B). Therefore, preventive measures such as improvements in sanitation, rodent control, and proper waste management are essential to reducing the incidence of the disease. Rapid urbanization and unplanned growth has resulted in the occupation of environmentally sensitive areas, increasing the exposure of vulnerable communities to floods and the risk of leptospirosis [38]. In Campinas, these situations, combined with the presence of rivers such as the Atibaia and Capivari, have become a recurring event, especially during the rainy summer season (December to March). The epidemiological data showed a significant increase in leptospirosis cases following periods of heavy rainfall, where low-income regions are more susceptible to flooding, and consequently, leptospirosis outbreaks [39]. Particularly in extensive areas of Campinas, systematic processes of urban planning that lead to people being exposed to the hazard are inferred, with persistent floods, floodplains management and stormwater design being below optimal conditions [40], with numerous plans on paper, yet lacking alignment with real-world needs [41]. Plans for managing can also be funded by different government levels, but prefecture and state level funding of different infrastructure are not necessarily being conducted in an integrative way. Specifically for the eastern regions of Campinas (Cabras), it is not clear which standards are considered flood prevention for pedestrians. Therefore, the post-flood transmission risk of leptospirosis in Campinas remains a health problem since the last decades [39]. These risk areas for flood-prone leptospirosis cases are closely related to high social vulnerability (Fig 4B). More than half a million people (~60% of the total population of Campinas) live in areas of high and medium-high vulnerability and most of the population in these areas have an average income of between one and two minimum wages. These areas are more clustered in the floodplains of the Quilombo and Atibaia Rivers [42].

Environmental characteristics of urban areas such as populated urban slum areas that lack adequate water supply and sanitation [43] can significantly contribute to disease risk. Other key elements include habits and hygiene behavior, as well as ecosystem and pathogen-related factors. When these factors come together, they create a permeable proximity with the pathogen in the environment [14]. The difficulty in assessing the influence of environmental conditions on the rapid dissemination of infection is that these conditions vary at different time intervals and at different scales. The severity and frequency of rainfall, the compaction degree of the urban areas, and the quality of sanitary infrastructure are examples of circumstances which vary spatially and socially [15, 44]. Assessing these conditions can be more difficult in areas where flooding events are frequent. Finding consistent patterns in areas with a higher incidence of leptospirosis often coincide with regions of high population density and low urban infrastructure index [45] is useful for preparedness plans informed by spatial indexes and remotely sensed data [46]. Neighborhoods with specific characteristics, such as proximity to streams and irregular occupation of risk areas, with the highest levels of built areas denoting higher impermeability, present a higher concentration of cases. It is exacerbated in neighborhoods such as Campo Belo and Sousas, which often face seasonal floods that increase population exposure to contaminated water.

In Brazil, 10% of leptospirosis cases evolve to high severity. Leptospirosis remains a threat despite the recent decrease in the total number of cases, with the number of deaths breaking records in 2023 [47], and the outlook not looking positive for the 2024 flood season. For people at risk in areas that have precarious drainage infrastructure (such as Princesa d’Oeste, Amoreiras Avenue surroundings), it is essential for governance to prioritize the allocation of resources toward awareness campaigns and mitigation strategies aimed at reducing the impact of leptospirosis during flooding events.

In contexts similar to the one we investigated here, local authorities should consistently invest in education and early warning systems (such as the ones proposed for malaria combat [48, 49]) that may inform stakeholders about the areas associated with flooding and leptospirosis transmission, as well as the preventive measures they can take. Furthermore, fostering robust community networks is crucial for developing effective response plans to address flooding scenarios, which are projected to increase in the Global South [50]. These plans should include strategies for supporting individuals who may need to commute or find shelter when floods threaten their homes, ensuring that vulnerable populations have access to safe spaces and necessary resources during emergencies. By strengthening community resilience and enhancing awareness, governance can significantly mitigate the health risks associated with leptospirosis in flood-prone areas.

Climatic extremes [51] are driven by multidimensional environmental factors that in turn interact with other risk components influencing risk of transmission of leptospirosis [52]. Because extreme events can continuously disturb and affect urban regions (like the proposed ‘Joseph effect’ in Campinas) [53], mitigation action plans are probably more needed than ever. São Paulo State has been suffering from higher extreme climatic events clustering in shorter rainy seasons and longer periods of heatwaves and drought [54]. Retrospective analysis like the one we present are useful, especially when trends are pervasive like we see with leptospirosis and floods in Campinas. Post-flood cases are common in urban settings, where a patient can be exposed to multiple contaminated water sources in a very brief period of time [55]. There is a current gap in studies about urban drainage network adaptation to climate change [56]. In urban settings in Brazil, infected rats with a high prevalence of infection were found among all age classes indicating efficient transmission [2, 57]. Sustainable rodent management options should be explored [58]. Given the complexity of factors contributing to leptospirosis transmission during floods, preventive strategies must be multisectoral [59], involving sanitation, rodent control, and public awareness. In Campinas, Epidemiological Surveillance and Civil Defense play an essential role in the prevention and response to leptospirosis outbreaks during flood events. Measures such as strengthening drainage infrastructure, adequate solid waste management, and educational campaigns are essential to reduce population exposure to contaminated water. Actions like these have been implemented in Campinas, but effectiveness is limited by the need for broader investments in urban infrastructure and services [17].

Studies on the spatial dynamics of leptospirosis are essential for epidemiological surveillance, as they help identify spatial patterns, risk areas, and environmental factors influencing disease spread. GIS tools facilitate the visualization of case distributions and potential risk drivers, supporting targeted interventions and resource allocation. These approaches also aid in monitoring outbreaks, planning preventive measures, and improving responses to health emergencies by informing surveillance strategies and public policies. Spatial analysis can thus play a key role in reducing transmission, particularly in the highly vulnerable communities that are prone to be the most affected. For example, it can guide municipal contingency plans by mapping low-coverage, highly vulnerable areas where personal protective equipment and strategic drug stockpiles for post-exposure prophylaxis should be prioritized. Collectively, these strategies underscore the importance of coordinated, intersectoral action that integrates structural urban improvements with health interventions, enhancing resilience to flooding events and lowering the burden of leptospirosis.

### Study limitations

There are several limitations to conducting neglected disease research in Brazil. The data associated with patients in the SINAN system is often incomplete, particularly regarding exposure history, such as the probable site of infection, the history of contact with floodwater or mud, or other environmental factors. During data curation, we observed that the databases on the probable site of infection exhibited typographical errors or lacked complete information. For example, Among the 51 cases classified as other exposure risks, 32 had complete information, while 19 were excluded because the probable site of infection was not specified (cadastral map identifier code missing). Consequently, these cases could not be georeferenced, which reduced the number of cases available for spatial analysis.

Additionally, as reported here, the quality of the data collected and recorded in SINAN can vary depending on the training of healthcare professionals and the resources available and other limited enabling conditions across different regions. This can lead to errors in completing the notification forms and affect the accuracy of the information. Another limitation is the demand for other indicators, such as rodent density or an infestation index. Such information could improve the effectiveness of the associations between leptospirosis and exposure environments. Moreover, it is important to highlight that some environments not explicitly categorized as “mud contact” in the SINAN forms, such as vacant lots, crop fields, and animal husbandry sites may potentially involve exposure to mud. These areas are typically unpaved and can accumulate water and organic waste, especially during or after rainfall, irrigation, or animal activity, resulting in muddy soil conditions favorable to Leptospira survival. The absence of direct attribution to mud may underestimate the general exposure pathways. This limitation reinforces the need for complementary field investigations and broader environmental characterization when analyzing leptospirosis risk factors. However, these problems are not exclusive to leptospirosis records. Correia et al. [60] evaluated the completeness of Health Information Systems in Brazil, including SINAN, and identified heterogeneous levels of completeness and inconsistencies, particularly in essential epidemiological variables. These findings demonstrate that data limitations are a structural challenge of national surveillance systems and should be carefully considered when interpreting spatial analyses and drawing conclusions. Nevertheless, it is noteworthy that health databases with completeness scores above 80% can be classified as having good informational quality. In this context, the database used in this study presented a completeness level of 82%, placing it within an acceptable standard for epidemiological analyses. Therefore, the results obtained can be considered consistent and methodologically robust.

## Conclusion

This study underscores the spatial patterns of leptospirosis in Campinas, with a particular focus on how socio-environmental and climatic factors influence the disease’s risk. Proximity to drainage systems, impermeable surfaces, and high social vulnerability emerge as key determinants of case distribution, with flood-prone areas experiencing the greatest risk, which corroborates our hypothesis. Cases associated with floodwater and mud exposure are consistently more frequent in the most vulnerable communities, underscoring the inherently compounded risk posed by inadequate infrastructure and environmental conditions. The increase in extreme weather events worsens the risk of contamination by increasing the frequency of floods, emphasizing the need for effective preventive measures and public policies. The study suggests that improvements in urban infrastructure, including drainage systems, access to basic sanitation and flood response systems, could significantly reduce cases of leptospirosis particularly in the Global South. Furthermore, collaboration between different government sectors, such as health, environment, and urban planning, remains essential to mitigate the impacts of floods and control the transmission of the disease. The study also highlights the importance of educational campaigns to raise awareness about the risks of leptospirosis and protective measures during flood periods. The methods used can be applied to other cities with similar socioeconomic and environmental contexts, aiding in the generation of evidence that could inform the development of public health policies aimed at reducing leptospirosis.

## Supporting information

S1 Fig Spatial distribution of rainfall events from 2007 to 2010 that caused flooding events in Campinas, São Paulo, southeastern Brazil. Administrative limits: https://informacao-didc.campinas.sp.gov.br/exporta_shp.php?id=119 (accessed March 2025). The data is free of licenses complying with the Transparência Pública Brasil (https://www.gov.br/cgu/pt-br/centrais-de-conteudo/campanhas/integridade-publica/transparencia-publica). Note that this data comes from Brazilian Public websites that may limit access to IP addresses outside of Brazil.
(TIF)

S2 Fig Confirmed leptospirosis cases in Campinas, southeastern Brazil, between 2001 and 2023. (TIF)

S1 Table Data on the frequency of leptospirosis cases who reported contact with floodwater/mud and other kinds of exposure from 2007 to 2010 according to distance from urban drainage. (XLSX)

S2 Table Density of leptospirosis cases who reported contact with floodwater/mud and other kinds of exposure from 2007 to 2010 according to social vulnerability levels. (XLSX)

S3 Table Number of leptospirosis cases who reported contact with floodwater/mud and other types of exposure from 2007 to 2010 according to the impervious surfaces index. (XLSX)

## References

[1] Hartinger SM, Palmeiro-Silva YK, Llerena-Cayo C, Blanco-Villafuerte L, Escobar LE, Diaz A, et al. The 2023 Latin America report of the Lancet Countdown on health and climate change: the imperative for health-centred climate-resilient development. Lancet Reg Health Am. 2024;33:100746. doi: 10.1016/j.lana.2024.100746 38800647 PMC11117061

[2] Costa F, Wunder EA Jr, De Oliveira D, Bisht V, Rodrigues G, Reis MG, et al. Patterns in Leptospira Shedding in Norway Rats (Rattus norvegicus) from Brazilian Slum Communities at High Risk of Disease Transmission. PLoS Negl Trop Dis. 2015;9(6):e0003819. doi: 10.1371/journal.pntd.0003819 26047009 PMC4457861

[3] Lau CL, Smythe LD, Craig SB, Weinstein P. Climate change, flooding, urbanisation and leptospirosis: fuelling the fire?. Trans R Soc Trop Med Hyg. 2010;104(10):631–8. doi: 10.1016/j.trstmh.2010.07.002 20813388

[4] Alied M, Salam A, Sediqi SM, Kwaah PA, Tran L, Huy NT. Disaster after disaster: the outbreak of infectious diseases in Pakistan in the wake of 2022 floods. Ann Med Surg (Lond). 2023;86(2):891–8. doi: 10.1097/MS9.0000000000001597 38333326 PMC10849431

[5] Nurhasana R, Hartono RK. The risk of non-communicable diseases after being exposed to the urban flood; a literature review and meta-analysis. IJG. 2021;53(3). doi: 10.22146/ijg.65401

[6] Soo ZMP, Khan NA, Siddiqui R. Leptospirosis: Increasing importance in developing countries. Acta Trop. 2020;201:105183. doi: 10.1016/j.actatropica.2019.105183 31542372

[7] Karpagam KB, Ganesh B. Leptospirosis: a neglected tropical zoonotic infection of public health importance-an updated review. Eur J Clin Microbiol Infect Dis. 2020;39(5):835–46. doi: 10.1007/s10096-019-03797-4 31898795

[8] Munoz-Zanzi C, Groene E, Morawski BM, Bonner K, Costa F, Bertherat E. A systematic literature review of leptospirosis outbreaks worldwide, 1970-2012. Rev Panam Salud Publica. 2020;44:e78. 10.26633/RPSP.2020.78 PMC7363284 32684917

[9] Pinto GV, Senthilkumar K, Rai P, Kabekkodu SP, Karunasagar I, Kumar BK. Current methods for the diagnosis of leptospirosis: Issues and challenges. J Microbiol Methods. 2022;195:106438. doi: 10.1016/j.mimet.2022.106438 35248601

[10] Dumitrescu C, Haber EN, Jamieson BD. Leptospirosis causing pulmonary hemorrhage and multiorgan system failure. Chest. 2022;162(4):A945. doi: 10.1016/j.chest.2022.08.744

[11] Galdino GS, Bezerra da Silva Junior G, De Francesco Daher E. Leptospirosis and Weil’s Syndrome. Tropical Nephrology. Cham: Springer International Publishing. 2020. p. 263–76.

[12] Talukder H, Muñoz-Zanzi C, Salgado M, Berg S, Yang A. Identifying the Drivers Related to Animal Reservoirs, Environment, and Socio-Demography of Human Leptospirosis in Different Community Types of Southern Chile: An Application of Machine Learning Algorithm in One Health Perspective. Pathogens. 2024;13(8):687. doi: 10.3390/pathogens13080687 39204287 PMC11357164

[13] Picardeau M. Virulence of the zoonotic agent of leptospirosis: still terra incognita?. Nat Rev Microbiol. 2017;15(5):297–307. doi: 10.1038/nrmicro.2017.5 28260786

[14] Bradley EA, Lockaby G. Leptospirosis and the Environment: A Review and Future Directions. Pathogens. 2023;12(9):1167. doi: 10.3390/pathogens12091167 37764975 PMC10538202

[15] Suwannarong K, Soonthornworasiri N, Maneekan P, Yimsamran S, Balthip K, Maneewatchararangsri S, et al. Rodent-Human Interface: Behavioral Risk Factors and Leptospirosis in a Province in the Central Region of Thailand. Vet Sci. 2022;9(2):85. doi: 10.3390/vetsci9020085 35202338 PMC8878075

[16] Thibeaux R, Genthon P, Govan R, Selmaoui-Folcher N, Tramier C, Kainiu M, et al. Rainfall-driven resuspension of pathogenic Leptospira in a leptospirosis hotspot. Sci Total Environ. 2024;911:168700. doi: 10.1016/j.scitotenv.2023.168700 37992819

[17] Costa F, Hagan JE, Calcagno J, Kane M, Torgerson P, Martinez-Silveira MS, et al. Global Morbidity and Mortality of Leptospirosis: A Systematic Review. PLoS Negl Trop Dis. 2015;9(9):e0003898. doi: 10.1371/journal.pntd.0003898 26379143 PMC4574773

[18] Schneider MC, Najera P, Pereira MM, Machado G, dos Anjos CB, Rodrigues RO, et al. Leptospirosis in Rio Grande do Sul, Brazil: An Ecosystem Approach in the Animal-Human Interface. PLoS Negl Trop Dis. 2015;9(11):e0004095. doi: 10.1371/journal.pntd.0004095 26562157 PMC4643048

[19] Ahern M, Kovats RS, Wilkinson P, Few R, Matthies F. Global health impacts of floods: epidemiologic evidence. Epidemiol Rev. 2005;27:36–46. doi: 10.1093/epirev/mxi004 15958425

[20] Cunha M, Costa F, Ribeiro GS, Carvalho MS, Reis RB, Nery N Jr, et al. Rainfall and other meteorological factors as drivers of urban transmission of leptospirosis. PLoS Negl Trop Dis. 2022;16(4):e0007507. doi: 10.1371/journal.pntd.0007507 35404948 PMC9022820

[21] de Freitas CM, Ximenes EF. Floods and public health: a review of the recent scientific literature on the causes, consequences and responses to prevention and mitigation. Cien Saude Colet. 2012;17(6):1601–15. doi: 10.1590/s1413-81232012000600023 22699650

[22] Vega-Corredor MC, Opadeyi J. Hydrology and public health: linking human leptospirosis and local hydrological dynamics in Trinidad, West Indies. Earth Perspectives. 2014;1(1). doi: 10.1186/2194-6434-1-3

[23] Brasil. IBGE Cidades. [cited 25 Nov 2024]. Available from: https://cidades.ibge.gov.br/

[24] Köppen W. Die Wärmezonen der Erde, nach der Dauer der heissen, gemässigten und kalten Zeit und nach der Wirkung der Wärme auf die organische Welt betrachtet. Meteorol Z. 1884;1:215–26.

[25] Beck HE, Zimmermann NE, McVicar TR, Vergopolan N, Berg A, Wood EF. Present and future Köppen-Geiger climate classification maps at 1-km resolution. Sci Data. 2018;5:180214. doi: 10.1038/sdata.2018.214 30375988 PMC6207062

[26] Datasus. In: SINAN: Doencas e agravos de notificacao. [Internet]. 2025 [cited 12 Jun 2025]. Available from: https://datasus.saude.gov.br/acesso-a-informacao/doencas-e-agravos-de-notificacao-de-2007-em-diante-sinan/

[27] Brasil. Leptospirose. In: Ministério da Saúde, Brasil [Internet]. 2024 [cited 10 Dec 2024]. Available from: https://www.gov.br/saude/pt-br/assuntos/saude-de-a-a-z/l/leptospirose

[28] R Core Team. R: A Language and Environment for Statistical Computing. Vienna, Austria: R Foundation for Statistical Computing; 2020. Available from: https://www.R-project.org/

[29] Wickham H. ggplot2: Elegant Graphics for Data Analysis. 2016. Available from: https://ggplot2.tidyverse.org/

[30] Campinas. Plano municipal de saneamento básico. Campinas: Prefeitura Municipal de Campinas. 2013.

[31] QGIS Development Team. QGIS Geographic Information System. Open Source Geospatial Foundation; 2009. Available from: http://qgis.osgeo.org

[32] Farias A, Mendonça F. Riscos socioambientais de inundação urbana sob a perspectiva do Sistema Ambiental Urbano. SN. 2022;34(1). doi: 10.14393/sn-v34-2022-63717

[33] Yamamoto JK, Landim PMB. Geoestatística: conceitos e aplicações. São Paulo: Oficina de Textos. 2013.

[34] São Paulo, Fundação Sistema Estadual de Análise de Dados - SEADE Índice Paulista de Vulnerabilidade Social (IPVS) - 2010. São Paulo: Governo do Estado de São Paulo, 2018. http://www.seade.gov.br/ [cited 2024 Oct 5]. Available from:

[35] USGS-U.S. Geological Survey. EarthExplorer. In: USGS - U.S. Geological Survey [Internet]. [cited 25 Nov 2024]. Available: https://earthexplorer.usgs.gov/

[36] Zha Y, Gao J, Ni S. Use of normalized difference built-up index in automatically mapping urban areas from TM imagery. International Journal of Remote Sensing. 2003;24(3):583–94. doi: 10.1080/01431160304987

[37] Carvalho-Pereira T, Eyre MT, Zeppelini CG, Espirito Santo VF, Santiago DC, Santana R, et al. Basic urban services fail to neutralise environmental determinants of ‘rattiness’, a composite metric of rat abundance. Urban Ecosyst. 2023;27(3):757–71. doi: 10.1007/s11252-023-01481-2

[38] Mwachui MA, Crump L, Hartskeerl R, Zinsstag J, Hattendorf J. Environmental and Behavioural Determinants of Leptospirosis Transmission: A Systematic Review. PLoS Negl Trop Dis. 2015;9(9):e0003843. doi: 10.1371/journal.pntd.0003843 26379035 PMC4574979

[39] Lara JM, Von Zuben A, Costa JV, Donalisio MR, Francisco PMSB. Leptospirosis in Campinas, São Paulo, Brazil: 2007-2014. Rev Bras Epidemiol. 2019;22:e190016. 10.1590/1980-549720190016 30942326

[40] Young AF, Jorge Papini JA. How can scenarios on flood disaster risk support urban response? A case study in Campinas Metropolitan Area (São Paulo, Brazil). Sustainable Cities and Society. 2020;61:102253. doi: 10.1016/j.scs.2020.102253

[41] Campinas. Plano Municipal de Saneamento Básico Produto 1. Diagnóstico Inicial. Capítulo 7: Drenagem e manejo das águas pluviais urbanas. Revisão 2012-2023. Prefeitura Municipal de Campinas; 2023. Available from: https://portal-api.campinas.sp.gov.br/sites/default/files/secretarias/arquivos-avulsos/142/2023/07/05-121719/p1-diagnostico-7%20-%20Drenagem.pdf

[42] Marques ML, da Silva MC, de Camargo DM. Análise espacial da vulnerabilidade socioambiental no município de Campinas, SP, Brasil. Revista Brasileira de Cartografia. 2017;69:1857–70.

[43] WHO. Preventing Disease through Healthy Environments: A Global Assessment of the Environmental Burden of Disease. World Health Organization. 2016. [cited 2025 Oct 5]. Available from: https://www.who.int/publications/i/item/9789241565196

[44] Teles AJ, Bohm BC, Silva SCM, Bruhn FRP. Socio-geographical factors and vulnerability to leptospirosis in South Brazil. BMC Public Health. 2023;23(1):1311. doi: 10.1186/s12889-023-16094-9 37420253 PMC10329394

[45] de Souza KOC, Góes JAP, Melo MS, Leite PMG, Andrade LA, Góes MA de O, et al. Spatiotemporal clustering, social inequities and the risk of leptospirosis in an endemic area of Brazil: a retrospective spatial modelling. Trans R Soc Trop Med Hyg. 2021;115(8):854–62. doi: 10.1093/trstmh/traa128 33205148

[46] Olivatto TF, Inguaggiato FF, Stanganini FN. Urban mapping and impacts assessment in a Brazilian irregular settlement using UAV-based imaging. Remote Sensing Applications: Society and Environment. 2023;29:100911. doi: 10.1016/j.rsase.2022.100911

[47] Globo São Paulo. Com sete mortes por leptospirose no ano, região de Campinas tem maior letalidade desde 2007. In: G1 [Internet]. 7 Dec 2023 [cited 25 Oct 2024]. Available from: https://g1.globo.com/sp/campinas-regiao/noticia/2023/12/07/com-sete-mortes-por-leptospirose-no-ano-regiao-de-campinas-tem-maior-letalidade-desde-2007.ghtml

[48] Gonzalez-Daza W, Vivero-Gómez RJ, Altamiranda-Saavedra M, Muylaert RL, Landeiro VL. Time lag effect on malaria transmission dynamics in an Amazonian Colombian municipality and importance for early warning systems. Sci Rep. 2023;13(1):18636. doi: 10.1038/s41598-023-44821-0 37903862 PMC10616112

[49] Srimokla O, Pan-Ngum W, Khamsiriwatchara A, Padungtod C, Tipmontree R, Choosri N, et al. Early warning systems for malaria outbreaks in Thailand: an anomaly detection approach. Malar J. 2024;23(1):11. doi: 10.1186/s12936-024-04837-x 38191421 PMC10775623

[50] Hirabayashi Y, Tanoue M, Sasaki O, Zhou X, Yamazaki D. Global exposure to flooding from the new CMIP6 climate model projections. Sci Rep. 2021;11(1):3740. doi: 10.1038/s41598-021-83279-w 33580166 PMC7881105

[51] Brunner MI. Floods and droughts: a multivariate perspective. Hydrol Earth Syst Sci. 2023;27(13):2479–97. doi: 10.5194/hess-27-2479-2023

[52] Mayfield HJ, Smith CS, Lowry JH, Watson CH, Baker MG, Kama M, et al. Predictive risk mapping of an environmentally-driven infectious disease using spatial Bayesian networks: A case study of leptospirosis in Fiji. PLoS Negl Trop Dis. 2018;12(10):e0006857. doi: 10.1371/journal.pntd.0006857 30307936 PMC6198991

[53] Bren L. Climate change, drought, and forest hydrology. Forest hydrology and catchment management. 2023. p. 329–57.

[54] Dunn RJH, Herold N, Alexander LV, Donat MG, Allan R, Bador M. Observed global changes in sector-relevant climate extremes indices—an extension to HadEX3. Earth and Space Science. 2024;11:e2023EA003279.

[55] Agampodi SB, Dahanayaka NJ, Bandaranayaka AK, Perera M, Priyankara S, Weerawansa P, et al. Regional differences of leptospirosis in Sri Lanka: observations from a flood-associated outbreak in 2011. PLoS Negl Trop Dis. 2014;8(1):e2626. doi: 10.1371/journal.pntd.0002626 24454971 PMC3894175

[56] Kourtis IM, Tsihrintzis VA. Adaptation of urban drainage networks to climate change: A review. Sci Total Environ. 2021;771:145431. doi: 10.1016/j.scitotenv.2021.145431 33736174

[57] Soni N, Eyre MT, Souza FN, Diggle PJ, Ko AI, Begon M, et al. Disentangling the influence of reservoir abundance and pathogen shedding on zoonotic spillover of the Leptospira agent in urban informal settlements. Front Public Health. 2024;12:1447592. doi: 10.3389/fpubh.2024.1447592 39360250 PMC11445007

[58] Stuart AM, Jacob J, Awoniyi AM, Costa F, Bosma L, Meheretu Y, et al. Alternative domestic rodent pest management approaches to address the hazardous use of metal phosphides in low- and middle-income countries. J Pest Sci. 2024;98(1):89–111. doi: 10.1007/s10340-024-01825-7

[59] Sykes JE, Haake DA, Gamage CD, Mills WZ, Nally JE. A global one health perspective on leptospirosis in humans and animals. J Am Vet Med Assoc. 2022;260(13):1589–96. doi: 10.2460/javma.22.06.0258 35895801

[60] Correia LO dos S, Padilha BM, Vasconcelos SML. Methods for assessing the completeness of data in health information systems in Brazil: a systematic review. Cien Saude Colet. 2014;19(11):4467–78. doi: 10.1590/1413-812320141911.02822013 25351313

