# Supplementary figures and images for: Leptospirosis in Campinas, Brazil: The interplay between drainage, impermeable areas, and social vulnerability
Source: PLoS Negl Trop Dis. 2025 Sep 29;19(9):e0013560. doi: 10.1371/journal.pntd.0013560 (PMC12571306; doi:10.1371/journal.pntd.0013560)

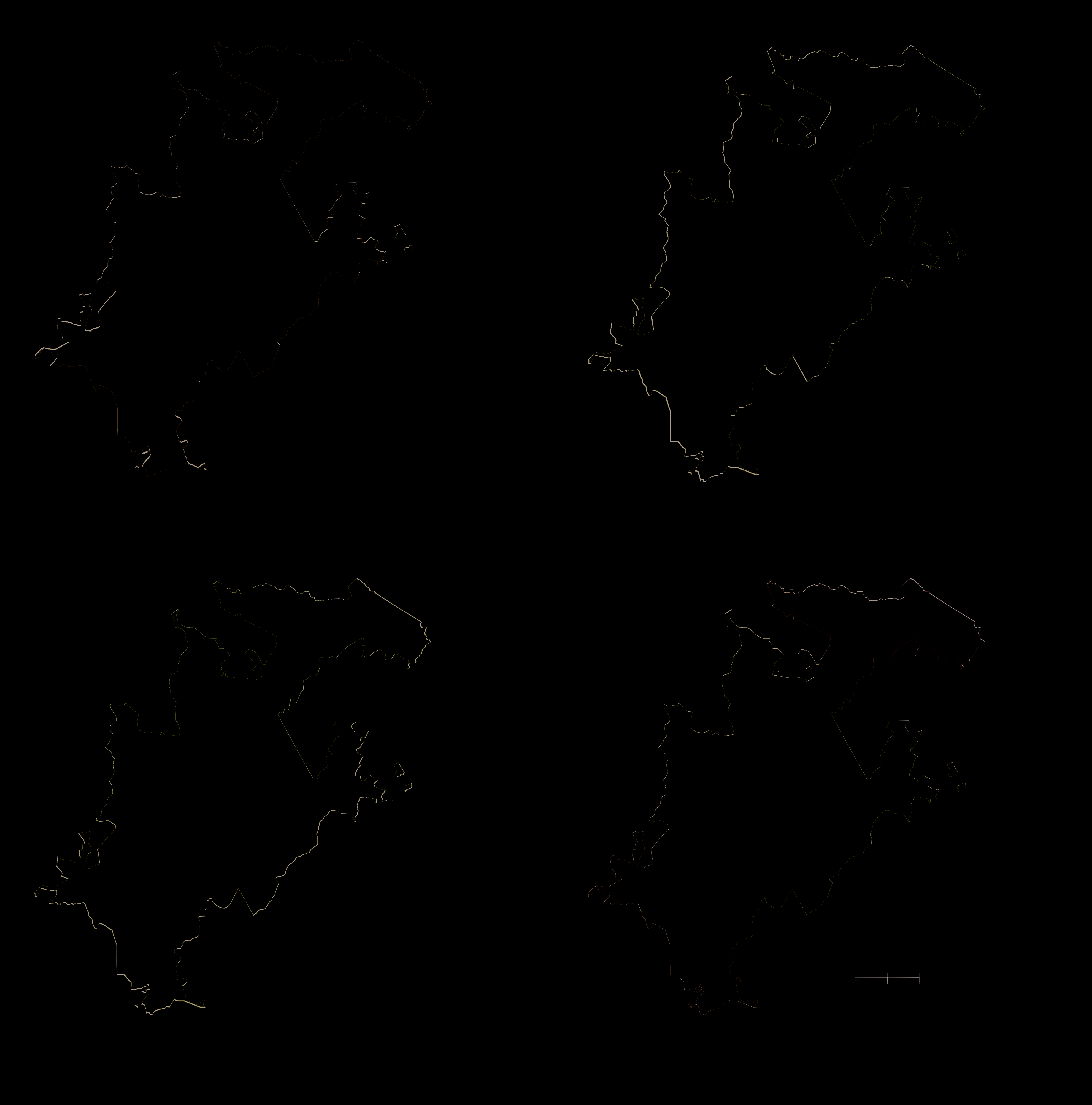

Supplement: S1 Fig — Administrative limits: https://informacao-didc.campinas.sp.gov.br/exporta_shp.php?id=119 (accessed March 2025). The data is free of licenses complying with the Transparência Pública Brasil (https://www.gov.br/cgu/pt-br/centrais-de-conteudo/campanhas/integridade-publica/transparencia-publica). Note that this data comes from Brazilian Public websites that may limit access to IP addresses outside of Brazil. (TIF) [file pntd.0013560.s001.tif]

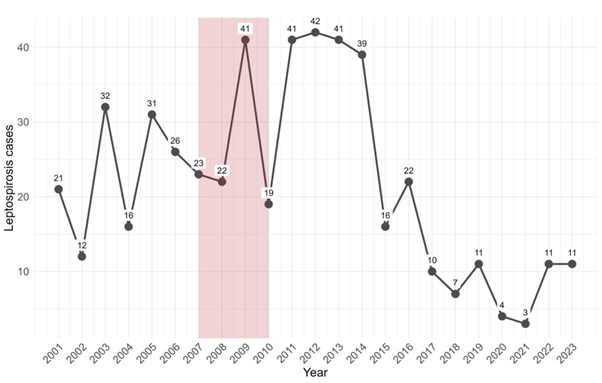

Supplement: S2 Fig — (TIF) [file pntd.0013560.s002.tif]
